# Applying the Stages of Change model to Type 2 diabetes care in Trinidad: A randomised trial

**DOI:** 10.1186/1477-5751-10-13

**Published:** 2011-10-11

**Authors:** VA Partapsingh, RG Maharaj, JM Rawlins

**Affiliations:** 1 Ste. Madeleine Health Centre, South-West Regional Health Authority, Trinidad and Tobago; 2 Unit of Public Health and Primary Care, Faculty of Medical Sciences, St. Augustine, The University of the West Indies, Trinidad and Tobago

## Abstract

**Objective:**

To improve glycaemic control among Type 2 diabetics using patient-physician consultations guided by the Stages of Change (SOC) model.

**Design and Methods:**

A randomised trial was conducted. After ensuring concealment of allocation, Type 2 diabetics were randomly assigned to receive the intervention or the control. The intervention consisted of identifying each patient's Stage of Change for managing their diabetes by diet, exercise and medications, and applying personalised, stage-specific care during the patient-physician consultations based on the SOC model. Patients in the control group received routine care. The variables of interest were effect on glycaemic control (measured by the difference in HbA_1c _levels) and patients' readiness to change (measured by identifying patients' SOC for managing their diabetes by diet, exercise and medications).

**Results:**

Participants were primarily over age 50, male and Indo-Trinidadian. Most had received only a primary school education and over 65% had a monthly income of $320 USD/month or less. Sixty-one Type 2 diabetics participated in each arm. Three patients were lost to follow-up in the intervention arm. After 48 weeks, there was an overall increase in HbA_1c _of 0.52% (SE 0.17) and 1.09% (SE 0.18) for both the intervention and control groups respectively. There was a relative reduction in HbA_1c _of 0.57% (95% CI 0.07, 1.07) with the intervention group compared to the control (*p *= 0.025). For exercise and diet there was an overall tendency for participants in the intervention arm to move to a more favourable SOC, but little change was noted with regards medication use.

**Conclusions:**

The result suggests a tendency to a worsening of glycaemic control in this population despite adopting more favourable SOC for diet and exercise. We hypothesized that harsh social conditions prevailing at the time of the study overrode the clinical intervention.

## Background

The Stages of Change model postulates behavioural change as a process of 5 identifiable stages through which patients pass: precontemplation, contemplation, preparation, action and maintenance [1-3]. The model illustrates that for most persons a change in behaviour occurs gradually, with the patient moving from being uninterested, unaware or unwilling to make a change (precontemplation), to considering a change (contemplation), or deciding and preparing to make a change (preparation); genuine, determined action is then taken and, over time, attempts to maintain the new behaviour occur [1]. This 'stage' concept allows for applying a temporal dimension to the Stages of Change [4]. Within the model, relapses are almost inevitable and become part of the process of working toward life-long change [1].

The Stages of Change model has been evaluated in a number of contexts [1,3,5], and although not widely used to provide care for Type 2 diabetes, it has been used to guide interventions for dietary change [5] and exercise behavior [1], both of which are important in managing diabetes. The intervention for this study was developed incorporating the Stages of Change model into the patient-physician consultation and attempted to answer the question: Does using the Stages of Change model to provide stage-specific and personalised care for managing Type 2 diabetes by diet, exercise and medications, improve glycaemic control in the Trinidadian setting?

## Methods

### The setting

Trinidad and Tobago has the sixth highest prevalence of diabetes mellitus in the Caribbean [6], affecting between 10 - 20% of adults, 85-90% of whom can be classified as Type 2 diabetics [7,8]. Care for these patients in the public health system in Trinidad and Tobago is provided by specialists at secondary and tertiary care centres and by primary care physicians at over 70 Primary Health Care (PHC) centres. This research was conducted at the Ste. Madeleine Health Centre (SMHC) in south Trinidad. At this clinic, care for Type 2 diabetes is offered through weekly sessions: the Chronic Disease (CD), Phlebotomy, Dietician, and Walk-in clinics. Approximately 50 patients are scheduled for each session with return visits in 16 weeks. The governing health authority requires that Walk-In clinic services be available everyday, all day. To facilitate this, the Walk-In clinic is conducted simultaneously with the CD clinic, using the same staff members. Two audits illustrate the limits of the present policy. The first, an audit of diabetic control suggested that mean HbA_1c _was 8.5%. Additionally an audit of patient-physician consultation time determined that 59% of consultations lasted less than 6 minutes and 38% lasted 4 minutes or less. International studies report average consultations in primary care to range from 7-10 minutes [9]. Compromising on consultation time for apparent efficiency may act to diminish patient autonomy and encourage medical paternalism by limiting discussion of patient values, alternative treatments, or the impact of therapy on the patient's overall life [10]. At the SMHC the current approach has resulted in an increasing numbers of Type 2 diabetics who can be described as 'frequent-visitors.'

### Study design

**Figure 1:**
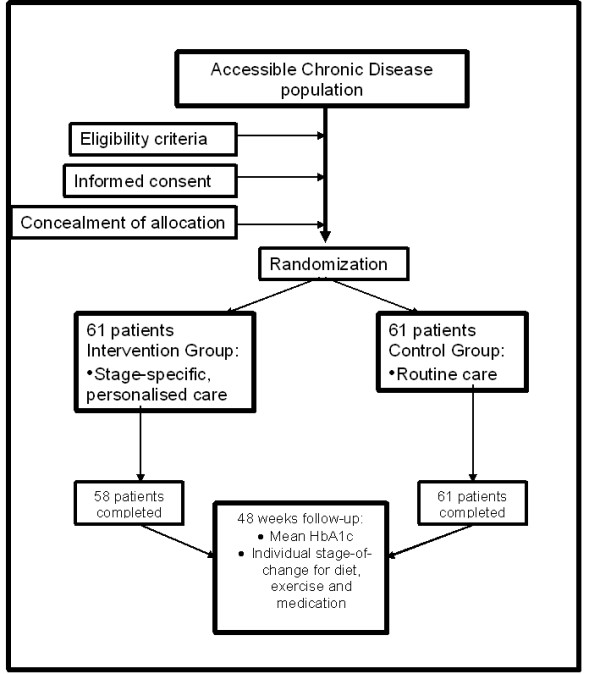
**Overview of study design**.

Approval for the conduction of the study was granted by the Ethics committee of the South West Regional Health Authority. Patients who fulfilled the inclusion and exclusion criteria were randomly allocated to either the control group or an intervention group. Over 48 weeks, subjects within the control group continued to receive routine care; subjects within the intervention group were treated with stage-specific, personalised care for Type 2 diabetes using the Stages of Change model. To ensure concealment of allocation, an independently designed randomisation schedule using a table of random numbers was created with sealed consecutively numbered opaque envelopes. Each envelope contained a card indicating either "Intervention" or "Control". See Figure 1.

### Sample size

Sample size was calculated using the formula [11]: Sample size (per equally-sized group) = 16 ÷ (E/S)^2^. A desired effect (E) size of HbA_1_c of 1% based on the results of the UKPDS [12] and an audit described above which provided a mean HbA_1_c of 8.48% and a standard deviation (S) of the mean of 1.94% gave a sample size of 61 patients in each arm.

### Inclusion and exclusion criteria

Patients were included if they were 69 years or less, were registered in the clinic's 'Chronic Disease Register' as Type 2 diabetics for the previous 12 months, not using insulin therapy at the time of recruitment and who agreed to participate after informed consent. Patients were excluded if they had plans to travel abroad for a period of more than four (4) weeks during the study, lacked decision-making capacity, or who were unable to perform activities of daily living. Additionally those who were receiving scheduled additional Type 2 diabetes care at any secondary or tertiary care center were also excluded. Once a patient was allocated to one of the treatment groups no other member of their household was eligible to participate in the study.

#### Outcome variables

##### Primary outcome variable

The primary outcome variable measured was HbA_1c _level. All HbA1c samples were tested at the same laboratory in the San Fernando hospital, Southwest Regional Health Authority in Trinidad. Other variables recorded included Body Mass Index, Blood pressure, plasma urea and creatinine, total cholesterol and triglycerides and random glucometer values.

##### Secondary outcome variable

Patients' readiness to change [1,2,4]

This represented patients' SOC [4] measured by identifying where patients are on the behaviour change process [1] for each of diet, exercise and management by medication.

All assessments were based on patient self-reported data. Action stage for exercise was defined as a person being involved in physical activity of moderate intensity, 3-5 days a week for at least 30-45 minutes per day. Action stage for medication use (for a person) was defined as that person adhering to their prescribed medication regimen. Action stage for diet was assessed based on a person reporting use of a specific dietary plan for managing diabetes.

### The intervention

The intervention was 'stage-specific' and personalised: it delivered care to Type 2 diabetics that was specific to the patient's current SOC and specific to the patient as a whole. These formats divided each consultation into sections specific for the named SOC. Each format was translated into a form which was used at each patient-physician consultation. There were five forms in this study and each patient was exposed to the one appropriate to their present SOC with respect to diet, exercise and medication use. These forms were used as checklists for the physician to ensure all the sections of the consultation were attended to during the visit. Examples of these forms are included in the appendix (See Additional File 1). Consultation times were not measured in this study.

### The control group

Patients in the control group continued with their routine care, this involved monitoring weight, blood glucose, blood pressure, discussing concerns with staff, if any recognised; and receiving a prescription for their appropriate medications.

### Follow-up and adherence to protocol

The total number of patients in the control and intervention groups were divided into four (4) subgroups and seen at four (4) consecutive CD clinic sessions and four (4) clinical sessions dedicated to the stage-specific personalized care for Type 2 diabetes patients, respectively. This grouping was maintained throughout the study. This pattern was repeated for each of the three 16-week cycles. Patients from either group who did not attend the SMHC for the following compulsory scheduled visits (initial and fourth (final) plus either of 2^nd ^or 3^rd ^visits) were not included in the statistical hypothesis testing.

## Results

All patients in the control group completed the study. Three (3) patients in the intervention group were lost to follow-up. The final analysis was performed with results from one hundred and nineteen (119) patients. Complete sets of measurements were available for one hundred and eighteen (118) of these patients. The greatest proportion of patients was between 40 to 59 years old; they were equally distributed in both groups: forty (66%) patients in the control and thirty-eight (62%) patients in the intervention group. Male patients constituted the greatest proportion of the research sample; thirty-nine (64%) patients of the intervention group and forty (66%) of the control group were males. Most patients in the study were of East Indian ethnicity (85% of the intervention group and 93% of the control group). More than half of the research sample had received only 7-8 years of formal education up to the end of primary school, forty-one (67%) patients in the control group, and thirty-five patients (57%) in the intervention group. Baseline characteristics are provided in Table 1 and results of the complete set of variables are presented in Table 2.

**Table 1:** Baseline characteristics of patients in the Intervention and Control groups

Descriptive characteristic		Number of patients N (%)	
		
		Intervention group 61 (100)	Control group 61 (100)
**Age**	20-39 years	6 (10)	2 (3)
	
	40-49 years	16 (26)	8 (13)
	
	50-59 years	22 (36)	32 (52)
	
	60-69 years	17 (28)	19 (31)

**Gender**	Male	39 (64)	40 (66)
	
	Female	22 (36)	21 (34)

**Ethnicity**	African	6 (10)	2 (3)
	
	East Indian	52 (85)	57 (93)
	
	Mixed	3 (5)	2 (3)

**Education level**	None	5 (8)	8 (13)
	
	Primary school (7-8 years duration)	35 (57)	41 (67)
	
	Secondary school	11 (18)	6 (10)
	
	Technical/vocational school	10 (16)	4 (7)
	
	University	0 (0)	2 (3)

**Employment status**	Retired	14 (23)	11 (18)
	
	Permanently employed	8 (13)	8 (13)
	
	Self-employed	8 (13)	9 (15)
	
	Occasionally employed/Unemployed	10 (17)	14 (23)
	
	Housewife only	21 (34)	19 (31)
	
	Pension or Government assistance	20 (34)	24 (40)

**Source of income**	Occupation	25 (41)	29 (48)
	
	Savings	0 (0)	2 (3)
	
	Spouse	14 (23)	19 (31)
	
	Children who are employed	6 (10)	12 (20)
	
	Other relative	1 (2)	0 (0)

**Total monthly income**	< $500.00	2 (3)	4 (7)
	
	< $1000.00	24 (39)	19 (31)
	
	< $2000.00	16 (26)	21 (34)
	
	> $2000.00	19 (31)	17 (28)

**Duration as diabetic**	1-5 years	27 (42)	16 (26)
	
	5-10 years	21 (34)	21 (34)
	
	> 10 years	13 (21)	24 (39)

**Existing co-morbid conditions**	Hypertension	31 (51)	34 (56)
	
	Ischaemic Heart Disease	5 (8)	9 (15)
	
	Hypercholesterolemia	23 (38)	26 (43)
	
	Asthma	0 (0)	1 (2)
	
	Osteoarthritis	2 (3)	1 (2)
	
	None	26 (43)	23 (38)

**Table 2:** Outcome variables and mean values at baseline and after 48 weeks for the intervention and control groups

Variable	Intervention	Group	Control	Group	*p-*value
	**Baseline** **Mean (SEM)** **N = 61**	**At 48 weeks** **Mean (SEM)** **N = 58**	**Baseline** **Mean (SEM)** **N = 61**	**At 48 weeks** **Mean (SEM)** **N = 61**	

**HbA**_**1**_**c (%)**	8.5 (0.3)	9.1 (0.29)	8.2 (0.28)	9.3 (0.29)	0.025

**Body Mass Index (kg/m2)**	29.1 (0.66)	28.8 (0.68)	27.7 (0.58)	27.8 (0.57)	*

**Systolic BP (mmHg)**	131.6 (2.32)	136.5 (2.43)	133.7 (2.44)	126.9 (1.92)	*

**Diastolic BP(mmHg)**	85.6 (1.41)	83.4 (1.81)	83.3 (1.2)	82.8 (0.93)	*

**Plasma Urea (mmol/l)**	14.3 (0.74)	15 (0.73)	16.6 (0.89)	16.4 (1.0)	*

**Plasma creatinine (mmol/l)**	1.0 (0.03)	1.0 (0.02)	1.0 (0.04)	1.0 (0.05)	*

**Fasting Total Cholesterol (mg/dl)**	214.3 (4.91)	214.4 (17.64)	232.7 (6.79)	217.2 (8.25)	*

**Fasting Total Triglycerides (mg/dl)**	156.8 (15.73)	152.9 (10.33)	190.7 (23.4)	195.6 (22.87)	*

**Random glucometer value (mg/dl)**	179.2 (9.06)	223.8 (11.12)	170.4 (9.54)	210.3 (7.73)	*

**Number of patients with albuminuria**	10 (NA)	5 (NA)	8 (NA)	6 (NA)	

**Number of hypoglycemic episodes**		0		0	

* There was no significant difference between the intervention and control groups with *p-*values > 0.05 for all variables except HbA_1_c as discussed.

### Results from primary outcome variable and statistical test of the hypothesis

The variable **"**Effect on glycaemic control" was the difference in glycaemic control (HbA_1c_) at the start of the study from that at the end of the study for both the intervention and control groups. The significance of this "Effect on glycaemic control" was tested (α of 0.05) using the independent-samples two-sided t-test. The result of the statistical test indicated the "Effect on glycaemic control" observed for the study was significant (*p *= 0.025): the intervention did improve glycaemic control when compared to the control group. From Table 2, glycaemic control worsened for both groups compared to baseline. The change in glycaemic control for the intervention group was a mean increase in HbA_1c _of 0.52% (SE 0.17) compared to that at baseline. The change in glycaemic control for the control group was a mean increase in HbA_1c _of 1.09% (SE 0.18) compared to that at baseline. The "Effect on glycaemic control" or the mean difference in change in HbA_1c _for the intervention from the control group is -0.57% (95%CI 0.07 - 1.07). For all other variables the statistical analysis gave *p*-values > 0.05.

### Results from secondary outcome variable - "Readiness to change"

**Figure 2:**
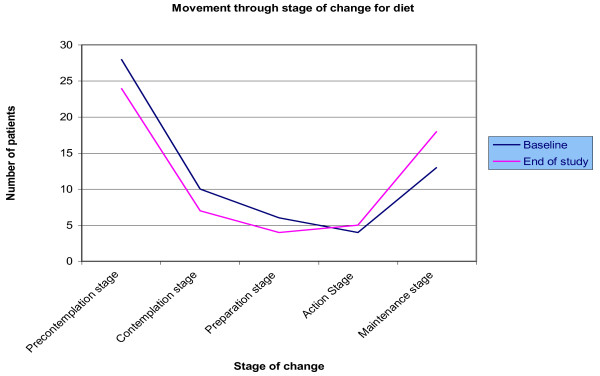
**Longitudinal comparisons of the Stage of Change shifts for the intervention group for Dietary behaviour**.

**Figure 3:**
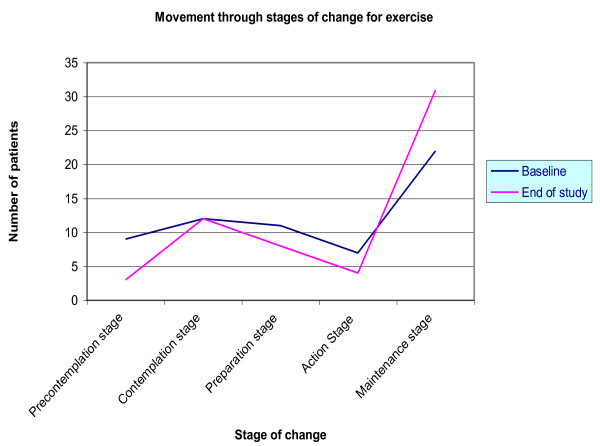
**Longitudinal comparisons of the Stage of Change shifts for the intervention group for Exercise behaviour**.

**Figure 4:**
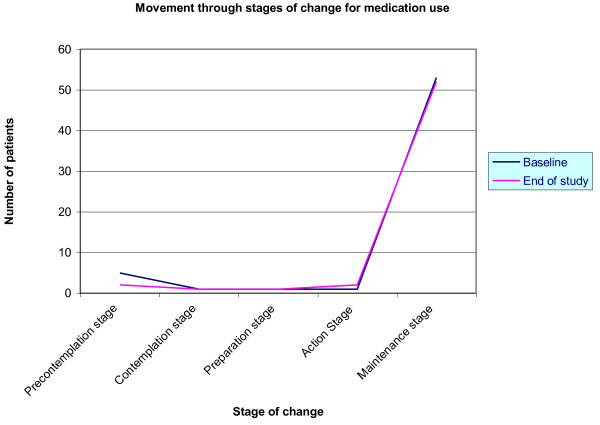
**Longitudinal comparisons of the Stage of Change shifts for the intervention group for Medication use**.

The largest number of patients to shift stages was observed in the intervention group for managing Type 2 diabetes by exercise: twenty-one (21) patients had moved positively and were more ready to change their exercise behaviour by the end of the study. A longitudinal comparison of the stage of change shifts for patients at the start of the study and at the end was performed and is illustrated in Figures 2, 3 and 4.

Among these twenty-one (21) patients: two (2) shifted from precontemplation to contemplation stage; three (3) patients shifted from being in the precontemplation stage to maintaining appropriate exercise behaviour for Type 2 Diabetes; four (4) patients were able to move to setting a date to start the appropriate exercise behaviour from initially being in the contemplation stage; four (4) patients each were able to move from preparation to action stage and preparation to maintenance stage; one (1) person moved from the contemplation to the maintenance stage; and three (3) patients continued the appropriate exercise behaviour throughout the study period.

From the chart for **dietary behaviour**, the slope of the line graphs illustrates that over the forty-eight (48) week study period, the number of patients in the precontemplation, contemplation and preparation stages decreased while the number of patients in the action and maintenance stages increased. For **exercise behaviour**, the pattern is slightly different: the number of patients in the precontemplation and preparation stages decreased and the numbers in the maintenance stage increased as for dietary behaviour, however, numbers in the contemplation stage show no change and the number in the action stage actually decreased. The line graphs for **medication use **indicate little or no change except for a decrease in the numbers of patients in the precontemplation stage.

Data on the number of patients who moved through the stages of change was collected for both the intervention and control groups. Fewer persons experienced a shift in SOC within the control group compared to the intervention group. Within the control group, the greatest number of positive shifts also occurred with change in exercise behavior: 4 persons moved from the action to maintenance stage, 1 person shifted from precontemplation to action stage, 1 person shifted from precontemplation to maintenance stage and 1 person from preparation to action stage.

## Discussion

The aim of the study to improve glycaemic control by the end of the 48-week period was not realized. Interestingly however, the hypothesis test indicated there was a statistically significant difference between the change in glycaemic control measured for the intervention and control groups. The results from mean HbA_1c _indicated that all patients had poorer control of their diabetes at the end of the study compared to baseline. The hypothesis test indicated patients who received the intervention had a significantly smaller increase in HbA_1c _levels by the end of the study than those who received the control. The statistical significance of this result adds another dimension to the overall negative result as follows: although both the intervention and control groups had poorer glycaemic control reflected by higher HbA_1c _levels compared to baseline, the increase in HbA_1c _measured at the end of the 48-week period was 0.57% less with the intervention compared to the control (*p *= 0.025).

### Implications of the study

The negative result obtained highlight two important considerations: first, there is some value to applying the SOC model to type 2 diabetes care as evidenced by the 0.57% less rise in HbA_1_c relative to the control group, and second, there is possibly a tendency for glycaemic control to worsen over time among patients at SMHC.

### What do we know about this topic so far?

A large RCT supports the findings of this paper which suggests that patients can be moved from one stage of change to another and that this can be beneficial [13]. The longitudinal comparisons of the stage of change shifts from Figures 2, 3 and 4 illustrated these patterns which add support to the intervention model and its theory. A crossover pattern [14] was observed wherein the number of patients in the precontemplation, contemplation and preparation stages (collectively) decreased while there was a simultaneous increase in the number of patients in the action and maintenance stages (collectively) at the end of the study.

In that publication application of a stage of change model based intervention resulted in a greater reduction of HbA1c than standard care, but this did not reach statistical significance [13]. So this paper adds to the literature by illustrating that a statistically significant difference between intervention and controls can be achieved (even though there are limits to our results as we saw above).

### What other factors may have caused this bilateral worsening of glycemic control in both intervention and control groups?

After dialogue with patients we postulate that severe economic stress and social hardship facing the patients who utilized the SMHC during the time of the study and contributed to the unusual results. This economic hardship occurred because of the closure of the sugar factory, Caroni (1975) Limited [15] which was the major employer in the Ste. Madeline area. This closure meant that study participants would not have had the financial wherewithal to fully carry out the planned behaviour change, since this would involve more expensive diets and time spent exercising. This study started in February 2006, 3 years after the closure of the sugar industry, and at a time where many of the planned social buffers had not yet been put in place.

### Limitations of the Stages of Change model to Type 2 diabetes care at SMHC

The Stages of Change model was devised based on observations of people giving up smoking - an addictive behaviour requiring complete cessation [16]. Smoking can be considered to have one common set of behaviour patterns as it is a single behaviour. Managing type 2 diabetes by diet, exercise and medication use needs to consider the interaction of three different behaviours, each having differing sets of patterns, and each impacting on glycaemic control.

It is possible that patients engaging in exercise and dietary behaviours can be viewed as proceeding through a continuous directional flow through steps beginning with initiating the behaviour, followed by continuing it, while constantly adapting it during the diabetes-disease trajectory. Each of these steps, in turn, can be considered to have their unique set of SOC, including the possibility of new stages, and have their unique aims. The intervention model did not incorporate such a complex view of these behaviours and therefore it is possible that this could have contributed to the results observed.

As we noted above there is a need for economic considerations in whether the model succeeds or not.

### Limitation of the study

Complete blinding at any level (single, double, triple) was not achieved in this study since the PI provided care to all patients- both the intervention and control group. The PI was aware of the limits placed on the study by his involvement in these steps and placed due care on extraction of information from notes and in care of patients to ensure his personal biases did not interfere with the conduct of the study. Ideally additional personnel should be involved but the structure of the health services clinic did not allow for this. We acknowledge that this is a serious, but not fatal, shortcoming of the study.

### Planning for the future

The overall results suggest the possibility of a tendency for glycaemic control to be naturally worsened over time at SMHC. This directs attention to other factors, additional to the nature or style of the patient-physician consultation, that are instrumental to the success of achieving improved glycaemic control among type 2 diabetes at SMHC. These factors can include external physical factors, external psychological factors and internal psychological factors [17].

## Conclusion

The intervention used in this study was unable to improve overall glycaemic control for patients at SMHC despite the statistical significance of the relative reduction in HbA_1c_. The importance of other factors, especially the socio-economic factors influencing glycaemic control at SMHC, has been highlighted. Additionally, the possibility of an inherent tendency for glycaemic control to be worsened at SMHC, due to the influence of these factors, creates a worrying situation at the centre. The negative results obtained from this study provide a focal point to continue the search for an appropriate intervention to effectively improve HbA_1c _at the centre.

## Competing interests

The authors declare that they have no competing interests.

## Authors' contributions

This work was carried out by VAP as a component of his Doctor of Medicine (DM) (Family Medicine) degree from The University of the West Indies. RGM and JMR were his academic supervisors during the process. VAP conceptualized the project and RGM and JMR provided guidance to the final protocol, design and implementation. VAP conducted the clinical component and collected the data. All authors read, contributed to, and approved the final document.

## Supplementary Material

Additional file 1 **Appendix**. There were five forms used in this study for recording patient information based on their current Stage of Change with respect to diet, exercise and medication use. These forms were used as checklists for the physician to ensure all the sections of the consultation were attended to during the visit. An Example of these forms is included here. Click here for file
