# Supplementary material for: Applying the Stages of Change model to Type 2 diabetes care in Trinidad: A randomised trial
Source: J Negat Results Biomed. 2011 Oct 11;10:13. doi: 10.1186/1477-5751-10-13 (PMC3219566; doi:10.1186/1477-5751-10-13)
Supplement: Additional file 1 — Appendix. There were five forms used in this study for recording patient information based on their current Stage of Change with respect to diet, exercise and medication use. These forms were used as checklists for the physician to ensure all the sections of the consultation were attended to during the visit. An Example of these forms is included here. [file 1477-5751-10-13-S1.DOC]

APPENDIX

**STAGE OF CHANGE ASSESSMENT**

(Please fill in appropriate box)

**-**

Patient’s HC NUMBER – CD NUMBER

1. Have you been doing any regular* exercise to control your blood sugar?

**1**  **No**

**1** **Yes**

If Yes: If No:

1. How long now have you been exercising 3. Do you intend or plan to start exercising?

regularly*?

1 **Yes**

**0** **No**

**1** >**6months**

**0** <**6months**

4. If Yes: How soon from today do you plan to

start exercising?

1**In the next 30 days**

0**In the next 6 months**

**EVALUATION:**

| Question # | **Stages of change** | | | | |
| --- | --- | --- | --- | --- | --- |
|  | **Precontemplation** | **Contemplation** | **Preparation** | **Action** | **Maintenance** |
| 1 | 0 | 0 | 0 | 1 | 1 |
|  |  |  |  | 0 | 1 |
| 3 | 0 | 1 | 1 |  |  |
| 4 |  | 0 | 1 |  |  |

This patient’s current stage of change is:

*Regular exercise to assist in controlling blood glucose was defined as “Involvement in physical activity of moderate intensity, 3-5 days a week for at least 30-45 minute^7”^

**Consultation format – Pre-contemplation stage**

*Remember the use of communication skills*

Nature of this consultation

Patient’s HC NUMBER –CD NUMBER

**IC**

1^ST^ REC 2^nd^ REC 3^rd^ REC

**AIM: To get patient to START THINKING about the need to use**

**Diet Exercise Medications**

**to control blood glucose**

**______________________________________________________________________________________**

**SECTION 1:** Welcome patient to the consultation.

**______________________________________________________________________________________**

**SECTION 2:**

1. Provide the patient with measured values for the following and compare them to the normal values.

| **measure** | **patient’s value** | **normal value** |
| --- | --- | --- |
| Glucose-meter reading |  | <180 mg/dl |
| Blood pressure |  | 130/80 mmHg |
| HbA_1c_ |  | <7% |
| BMI |  | <25kg/m2 |
| Total cholesterol level |  | <166mg/dl |
| Triglyceride level |  | <150 mg/dl |

**SECTION 3:**

1. Use the pateint’s values above to indicate which ones place him/her at risk for complications of diabetes. (Do this by expressing your concern; do not use scare tactics e.g. “I am concerned about your blood pressure value being so high”)
2. Identify moments, during this section to provide small bits of relevant education.

Consultation format –precontemplation stage continued:

SECTION 4: Engage the patient –

1. How do you view/ picture yourself now?

How do you view/picture yourself if you were to control your blood sugar?

1. Do you know someone who controls their blood sugar well? Provide an example of such a patient.

Discuss how that patient is in control of their health, live healthy lives free of the complications: amputations, sores/ulcers, kidney failure, eye disease.

1. Do you know someone who is not in control of their blood sugar? Provide an example of such a patient.

Discuss how that patient is out of control of their health and experience the complications; amputations, sores/ulcers. kidney failure, eye disease.

1. Have you thought that the other members in your family know of the ways you are not controlling your blood sugar?
2. Have you thought that they would do the same for their lives – making them at greater risk for DM?
3. How would you know if your diabetes is affecting your eyes or your kidneys?
4. Do you know that there are many things available to help you control your blood sugar? –

Identify: C-DAP plan, playground in the area, options for eating out.

Consultation format – precontemplation stage continued

**SECTION 5:**

1. Formulate realistic therapeutic goals with the patient to be reviewed at next visit.

| **Measure** | **Personalised therapeutic goal** | **Normal Value** |
| --- | --- | --- |
| Glucose-meter reading |  | <180 mg/dl |
| Blood pressure |  | 130/80 mmHg |
| HbA_1c_ |  |  |
| BMI |  |  |
| Total cholesterol level |  | <166mg/dl |
| *Triglyceride level* |  | <150 mg/dl |
| *Diet* |  | Follow advice |
| *Exercise* |  | >30 mins > 3 times/week |

**SECTION 6:**

1. Discuss changes to management plan with patient e.g. medication.

Consultation format – precontemplation stage continued.

**SECTION 7**: Attend to the patient’s other complaints identified by NA.

­­­­­­­­­­­

**SECTION 8**: Summarise the consultation –

1. The patient’s abnormal values from SECTION 1 that puts the patient at risk.
2. Reinforce the advantages to managing diabetes the patient described in SECTION 4.
3. Recall the realistic therapeutic goals identified in SECTION 5.
4. Other.
